# Ayahuasca: Uses, Phytochemical and Biological Activities

**DOI:** 10.1007/s13659-019-0210-5

**Published:** 2019-05-27

**Authors:** Edgar Antonio Estrella-Parra, Julio Cesar Almanza-Pérez, Francisco Javier Alarcón-Aguilar

**Affiliations:** 1 Laboratorio de Farmacología. Dpto. Ciencias de la Salud. Div. C.B.S. Universidad Autónoma Metropolitana, Unidad Iztapalapa. Av. San Rafael Atlixco No. 186, Col. Vicentina, 09340 Mexico CDMX, Mexico; 2 Laboratorio de Fitoquímica, Laboratorio de Farmacognosia, Laboratorio de Fisiología Vegetal, Unidad UBIPRO, FES-Iztacala, UNAM, Tlalnepantla de Baz, 54090 Mexico CDMX, Mexico

**Keywords:** Ayahuasca, *Banisteriopsis caapi*, *Psychotria viridis* – dimethyltryptamine, β-carbolines, Psychotherapy

## Abstract

**Abstract:**

Ayahuasca (caapi, yajé), is a psychoactive brew from the Amazon Basin region of South America traditionally considered a “master plant.” It is prepared as a decoction from *Banisteriopsis caapi* and *Psychotria viridis*, which it is thought that it stimulates creative thinking and visual creativity. Native healers of the Orinoco and Amazon basins have used traditionally ayahuasca as a healing tool for multiple purposes, particularly to treat psychological disorders in the patients, with some beneficial effects experimentally and clinically validated. Recently, several syncretic religions, as the “União de Vegetal” (UDV) group in Brazil, have been spread around the world. The use of ayahuasca has been popularized by internet and smart-shops, bringing the psychoactive substance to new highs, emerging new “ayahuasqueros.” Ayahuasca has alkaloids as β-carbolines and dimethyltryptamines, which inhibit the monoamine oxidase and active the 5-HT_2A_ (5-hydroxytryptamine) receptor, respectively, resulting in hallucinations in the users. Ayahuasca induces a psychedelic change in the anteroposterior coupling of the electrophysiological brain oscillations in humans. Traditional ayahuasca beverage is generating pharmacological, commercial and spiritual interest among the scientific community, government people, and different populations worldwide. The goal of this article is to report about the uses, chemistry and biological activities of ayahuasca.

**Graphical Abstract:**

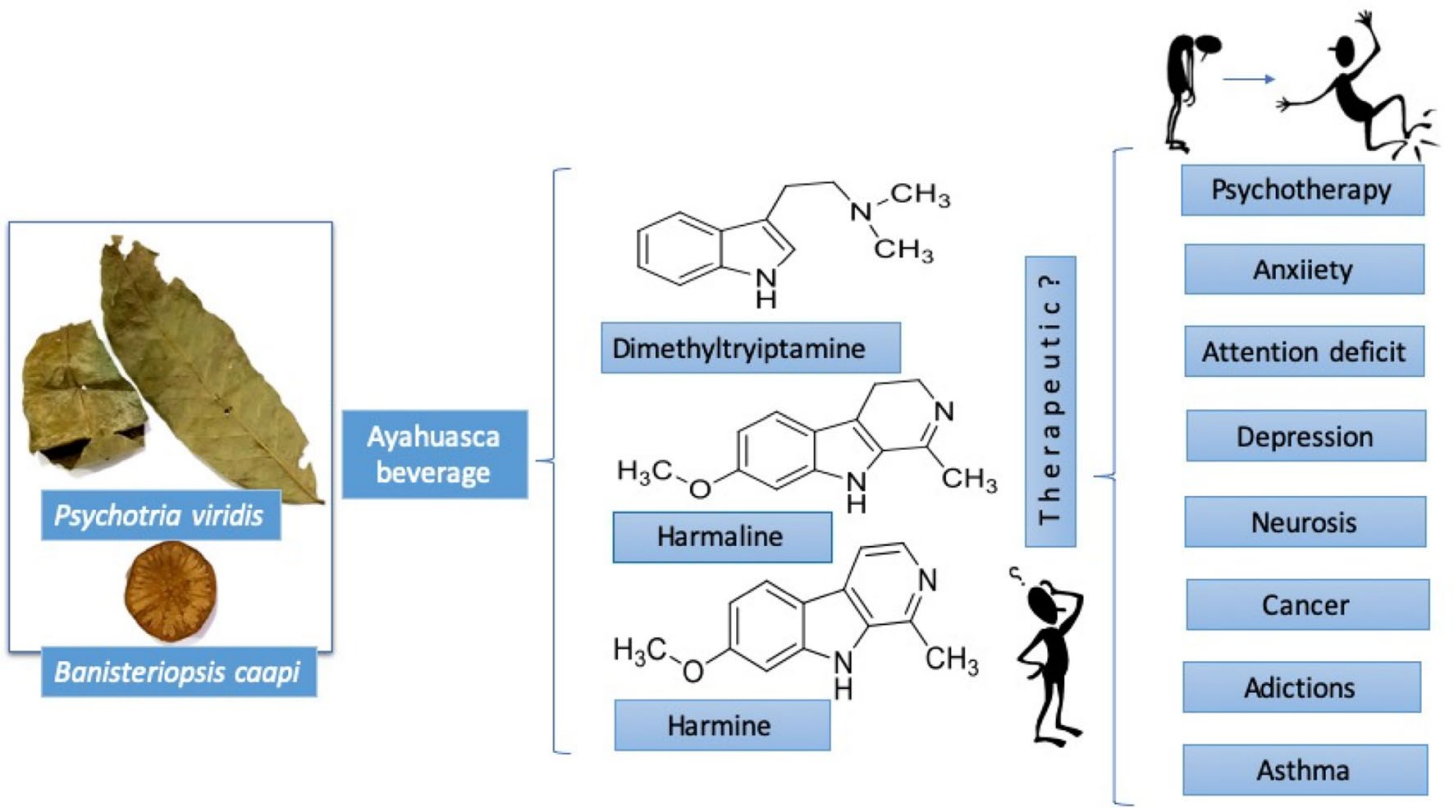

## Introduction

In the occidental world, the hallucinogens are psychoactive substances capable of inducing shifts in perception and feeling without a concomitant lapse of memory or loss of consciousness [1, 2]. Many compounds in plants and mushrooms as mescaline, dimethyltryptamine (DMT)(**1**), psilocybin, and lysergic acid produce these phenomenological effects [3]. The use of these hallucinogen plants and mushrooms in many communities of South America is a common practice. However, shaman Juan Mutumbajoy Jacanamijoy from Putumayo, Colombia, suggests these resources should be recognized as visualizers better than as hallucinogens (personal communication). According to his point of view, visualizer is a term that must be used to avoid the discrimination of the indigenous communities that use it, instead of the hallucinogen term.

The psychoactive substances have puzzled and fascinated humankind since its earliest days [4, 5]. Prue [6] mentioned two components as the cause of sickness (physiological and spiritual) and suggested that the therapy should include pharmaceutical and spiritual remedies [6]. Ayahuasca is a Quechua term that refers to a psychoactive preparation from the Amazon Basin region of South America, where it is considered one “master plant. In Colombia it is also called “caapi” or “yajé,” in Ecuador “Nate,” and Brazil “hoasca.” The etymology of the ayahuasca word in Quechua language coming of “aya” that meaning spirit (world of death, the other world) and “huasca” liana or vine (means rope), that in English may be traduced as “vine of the soul” [7].

**Fig. 1:**
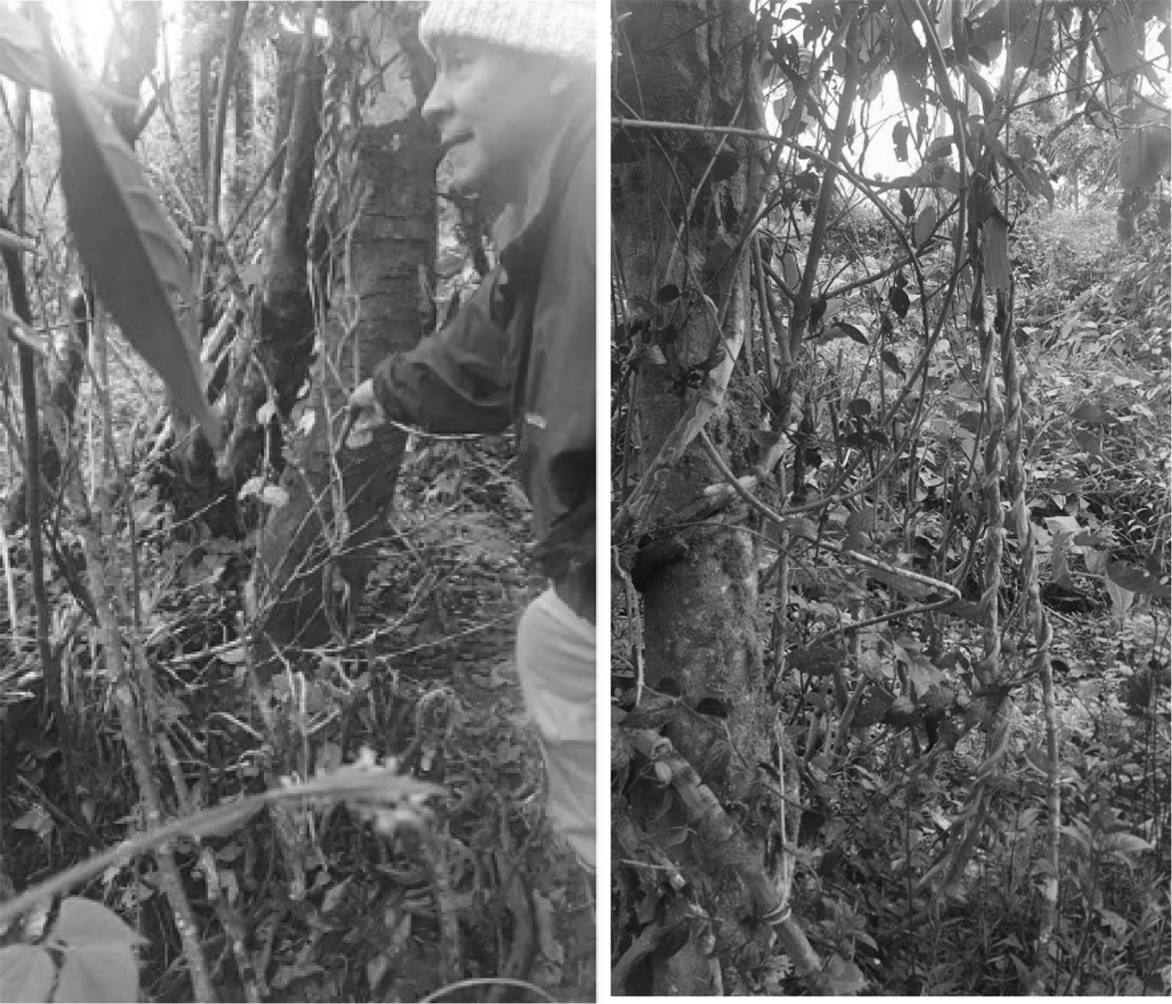
Shaman Juan Mutumbajoy Jacanamijoy showing the lianas of *Banisteriopsis caapi* (Malpighiaceae) at Vereda Tamabioy, Municipality of Sibundoy, Putumayo, Colombia

Ayahuasca beverage is prepared basically from the bark of the lianas *Banisteriopsis caapi* (Malpighiaceae) (Fig. 1) or *B. inebrians* with additives from some other species [8], mainly *Psychotria viridis* (Rubiaceae), popularly called chacruna, which has been used for many purposes by natives [8]. In the majority of the syncretic churches, ayahuasca beverage traditionally is prepared as follow: fragments (bark) of *B. caapi* are recollected and washed in water, pounded with a wooden mallet and carefully placed in a cauldron, alternating with washed leaves of *P. viridis.* Then water is added until the plant material is covered and the mixture is boiled and concentrated over at least 8 h to produce several liters. The resulting extract is basically dark. In the majority of the performed experimental and clinical studies is used a similar process of ayahuasca preparation, obtaining a decoction for oral administration (120 to 125 mL/patient) during rituals, according to the traditional practices of each region [9].

Ayahuasca reasonably used, it can be considered as a potent curative tool with beneficial effects validated. For instance, in several Latin American countries, like Peru, Brazil, Ecuador, Colombia, Argentina, Chile, and Mexico, ayahuasca has been used to treatment of addictions; however, its use also can entail risks [10]. The popularity of ayahuasca currently has impacted in a significant number of scientific publications in several areas, as sociological, psychological, psychiatric, neurologic, pharmacological, toxicological, and religious inclusive [11, 12]. The purpose of this review is to describe the uses, chemistry and pharmacological relevance of ayahuasca beverage.

## Origin

The origin of the use of the ayahuasca in the Amazon is poorly known [12]. Throughout the Amazon Basin, the use of ayahuasca remained profoundly rooted in tribal mythology and philosophy, that the modern investigators, as Anderson et al. [160] that defines the ayahuasca as a substance with transcendent pharmacological and personal implications [13], have concluded that its use extended back to the earliest aboriginal inhabitants of the region [14–16].

Several reports indicate that the scientific study of ayahuasca began with the English botanist Richard Spruce, who from 1849 to 1864 traveled extensively throughout the Brazilian, Venezuelan, and Ecuadorian Amazon to compile an inventory of the varieties of plant life found there [14, 15]. In general, ayahuasca is used by healers to treat psychological and physiological disorders in the patients [16], and after its ingestion they first suffer nausea, vomit or diarrhea [17].

**Fig. 2:**
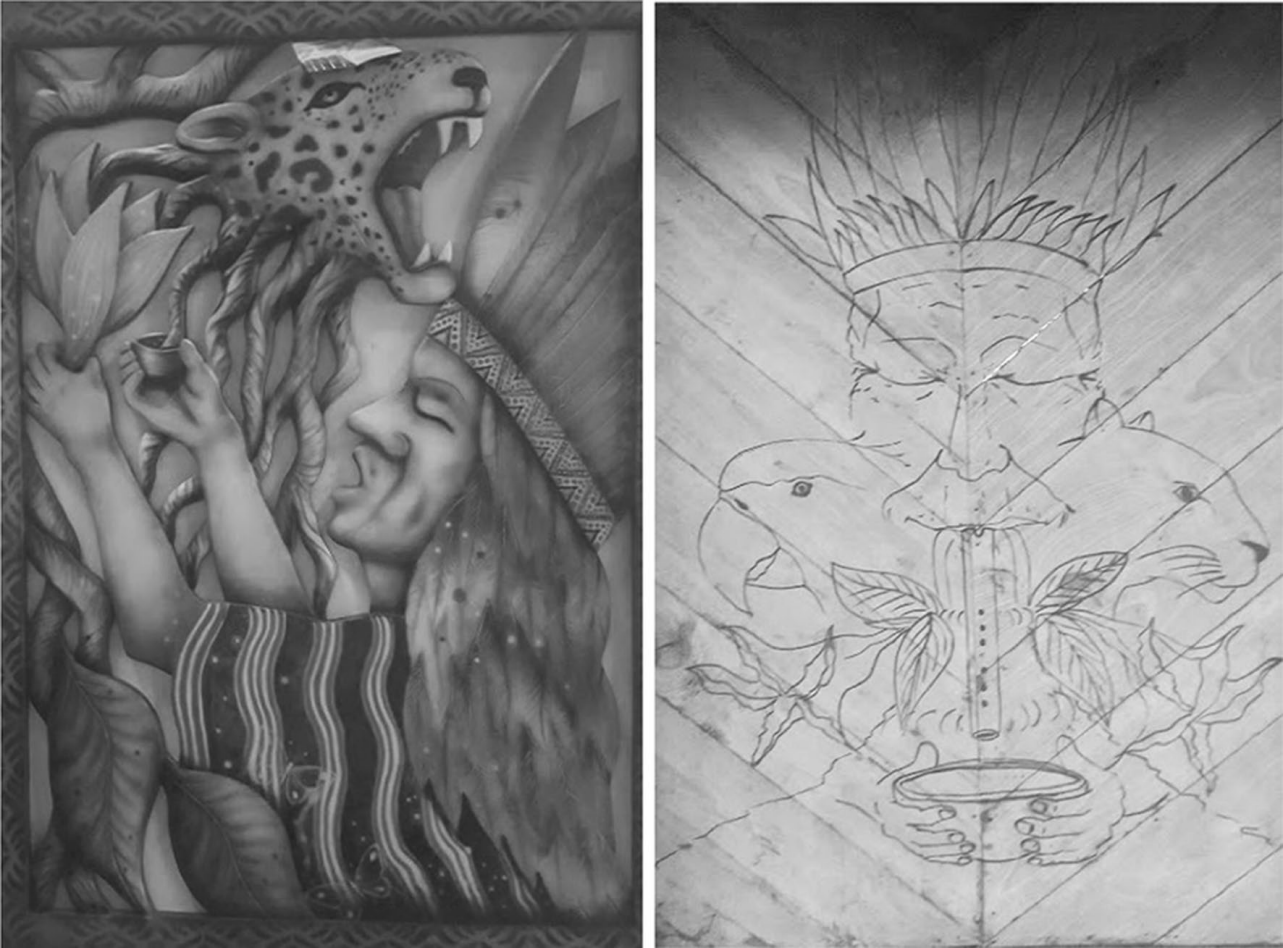
Pictures of Shaman in ayahuasca ritual referring to leaves of *P. viridis* and lianas of *B. caapi* in a vase containing ayahuasca beverage, often accompanied by singing, culminating in the vision of a jaguar or birds. Left: Picture at Ambiaku Turistic Center, Municipality de Colon, Putumayo, Colombia; Right: Wood carving in Maloka´s Shaman Juan Mutumbajoy at Vereda Tamabioy, Municipality of Sibundoy, Putumayo,Colombia

According to Ayala and Lewis [8], during the effects of ayahuasca, and depending on the dose administrated and of the susceptibility of the individual that ingest it, can experience different hallucination levels (visualizations) [8]. In the first level, the patient is questioned about its emotional problems; in the second level, he can begin to see faces of peoples with grotesque forms as well as other visualizations, including visions of animals (Fig. 2); also, there is light motor incoordination and nausea and vomit begin. In the end, the patient can experiment a sensation of flying while looks figures or spectacular sights with vivid colors, bright and intensified, through geometrical shapes, which become overt in the form of entopic images (within vision) maybe to recollecting past information [8].

The patient can fly, and eventually, gets back to the state of hallucination, ending exhausted from experience. If the patient takes ayahuasca over a long period, one last experience state of hallucination is called “telepath.” At this level, it is assumed that the patient may be able to illusory communicate with friends or family members (dead or alive) [8, 17].

The purgative effects of ayahuasca are often considered as a tonic rather than as toxic [9]. Daimistas, members of a Santo Daime doctrine that have used ayahuasca believe that the medicinal qualities of ayahuasca can uncover repressed emotions which are expelled as vomit or diarrhea [18]. It would be essential to carry out studies focused on determining the effects of ayahuasca on the possible changes in the microflora of the individuals who consume it and to study how these changes may impact the health, because gut microbiome can have a critical role in brain and behavior [19], and it also influences the central features of metabolic diseases [20, 21]. In this context, several medicinal (*Psacalium decompositum*) [22] and edible plants (*Opuntia ficus indica*) [23], some vegetal components, as morphine [24] or inclusive glycine [25–27], an inhibitory neurotransmitter with metabolic effects, have been proposed with impact on mental and metabolic diseases acting as modulators of gut microbiome. In particular, with ayahuasca, these aspects should be considered in further studies.

## Ayahuasca: A Master Plant

In a cultural context, ayahuasca beverage is traditionally recognized as a “master plant” that provide a framework for an encounter with the mythic, dramatic and psychological perspective of humans [28, 29]. According to this, master plants have been used to heal physical, emotional, spiritual imbalances as well as connecting with deep internal resonances [30], giving the user direct access to the spiritual world and to the storehouses of wisdom, which, otherwise, would have no access [5], facilitating existential intelligence [3, 18].

In the tradition of the Amazonian tribes, when warriors returned home from the hunting, the warrior drank ayahuasca to mitigate the aggression of hunting so they could reintegrate into the community [30, 31]. Today, In the United States and Europe, the use of ayahuasca has spread and is being used for self-analysis and the treatment of illnesses. It has become an ethnomedicine, which is thought to encompass all human dimensions [32]. It has been reported that the ayahuasca beverage is an example of cumulous effects of human experiences, where nature and man share the same space and time. Ayahuasca delivers a shot of imagination that it is not restricted to a place, but a metaphysical space [33].

Ayahuasca is regarded in many indigenous cultures as a powerful medicine for the body and the mind, promoting a harmonious relationship with others [33]. In this sense, ayahuasca may be understanding as a psycho-integrative plant, due to that integrate mind, body, spirit, and emotions in a safe, although socially sanctioned religious setting [2]. In short, ayahuasca is considered a physical, psychological and spiritual cleaning treatment whose consumption has been associated with welfare-related behaviors and with social and environmentally responsible comportments [34, 35], as observed Coral Herencia Vegas, a Peruvian archeologist and traditional therapist specialist in ayahuasca, who suggests that master plants must be considered within the sciences of consciousness (personal communication). In 2016, Soler et al. explored the potential therapeutic of ayahuasca, finding that its intake can increase these behaviors [35]. However, the psycho-integrative effects of ayahuasca are still poorly explored from a scientific perspective.

## Therapeutic Uses

Therapeutic uses of ayahuasca have been widely documented. For instance, in 1979 ayahuasca and harmaline (**7**) were used in 30 psycho-patients, of 10 exhibiting positive changes, in contrast with other intensive psychotherapy methods [1]. In Australian people, ayahuasca and DMT (**1**) induced cathartic effect and a smooth unusual psychic phenomenon, increasing psycho-spiritual insight [36]. In Colombia, inside the religious context, 40 people from Bogota city that consumed ayahuasca found inner peace and clarity to resolve complicated personal situations [37]. Also, ayahuasca in Brazilian teenagers reduced anxiety symptoms, less image dysmorphia, and improved attention deficit disorders [38], compared with teenagers that did not consume ayahuasca.

Soler [35], reported in 25 individuals that ayahuasca increases the mindfulness-related capacities [35]. Shanon [39], in an interview with around 200 ayahuasca users, deduced that the effects of this beverage should be associated to the human psyche, specifically with cognitive psychology, rather than to the brain and the culture. Agreed with this, the high state of mind induced by the psychoactive potion, suggests a non-ordinary state of consciousness that should be studied in further studies [40]. Besides, Tatjana [40] reported that 15 users that ingested ayahuasca during 1 year, who suffered different illness like cancer, asthma, depression, alcohol abuse, hepatitis C, migraine, fibromyalgia, and influenza, showed a better perspective of their lives and disease.

Winkelman [41] reported, in 11 people from European and North American, that ayahuasca enhances awareness of their self, access to deeper levels of the self, and personal growth [41]. Kuypers [42] reported in 26 Caucasian subjects, that ayahuasca enhances divergent creativity thinking, decreases fear, forgiven other/oneself, and promote self-acceptance; suggesting an increase in mental flexibility, and facilitating psychotherapeutic interventions to supporting clinical trial initiatives.

Even, the effect of ayahuasca in 17 gay and lesbian participants was explored, resulting in a re-define themselves in a positive way, improving self-acceptance and identity [43]. Finally, in an interview to Richard Yensen by Wolfson [44], mentioned its use in psychedelic psychotherapy for treating patients with substance abuse disorders, cancer, neurosis, and other illnesses; concluding that the psychedelic therapy induces safest, most home-like atmosphere: peaceful, unthreatening in the patients [44].

Concerning the use of ayahuasca as antiaddictive, in North America Santo Daime and “União de Vegetal” (UDV) groups, 81 ayahuasca users exhibited reduced alcohol intake, consuming a healthier diet, showing the improved mood and greater self-acceptance. Also, the subjects felt more loving and compassionate in their relationships [45]. Grob [14] reported in 15 members of the UDV syncretic church, the total abstinence from alcohol and other drugs due to ingestion of ayahuasca. The participants reduced the anger, resentments, and aggression as well as acquired better self-control and responsibility with their families [14].

In Brazil, ayahuasca user teenagers from the UDV group religion consumed less alcohol (32.5%) that those non-user adolescents of ayahuasca (65.1%), similarly to other psychoactive drugs as cannabis, amphetamines, cocaine, sedatives, tranquilizers, tobacco, among others drugs [46, 47]. Another example of ayahuasca as a detoxifying drug was with a Brazilian patient, who recovered her health after of consume alcohol, cocaine, and nicotine during at least 2 years, changing her attitude in the life [48].

All these different types of treatment by ayahuasca may exemplify the potential of this resource as psychedelic psychotherapy. To explain ayahuasca effect, it has been proposed that cures at “psycho-spiritual” level, rather than through physiological mechanisms [18]. Tatjana [40] also remarked that the use of ayahuasca should be interpreted as a psychological catalyst that unfolds within a field of sociocultural ideas and not only from a pharmacological perspective [40]. From this point of view, in further studies, both approaches should be considered.

## Uses of Ayahuasca as Psychedelic Psychotherapy

Around ayahuasca, there are different comments and experiences, in different cultures and countries. The reason why occidental people travel to South America to live the ayahuasca experience is curiosity, desire to treat mental health problems, need for self-knowledge, spiritual development, and finding direction in life [49]. Besides, people who have experienced ayahuasca exhibit positive, active, and ambitious behaviors toward life [50]. People from Northern Europe mentioned that ayahuasca use induced a positive psychological and physical improvements as a sense of being more present in oneself, in contrast, negative mental patterns as fear and abuse decreased [50]. In particular, the use of ayahuasca in the members of The Sant Daime church in the USA improved their mental clarity and sense of life purpose, as well as reduction of both anxiety and alcohol abuse [51].

There are many hypotheses to explain ayahuasca effects; however, in general, they were suggested from a subjective perspective that should be scientifically explored. For instance, Shanon [52], reported that the ayahuasca visions are described more real the real due to the sharp colors and feel of reality, with a closer approach from a spiritual perspective, resulting in one of the most significant experiences of life [52]. Other authors as Mabit [53], believe that the ingestion of ayahuasca stimulates the images in a context therapeutic symbolically manifest the content of their unconscious [53]. Trichter et al. [1] also reported that ayahuasca is useful in psychedelic illness, due to integrate and reveal the whole pattern behavior and the entire system of knowledge of an individual [1].

In this context, Loizaga-Velder mentions that ayahuasca is useful as a therapeutic tool, due to that acts as a catalyst that can render psychotherapeutic processes more effective in less time [54], facilitating interconnection among body-oriented, psychological and spiritual processes in the persons [8]. Inclusive Callaway [9], mentioned that the neurochemical agents of ayahuasca are potent tools that can enable a more comprehensive study of the mind [18]. From all this, McKenna [12] proposed to the ayahuasca as the “holy grail,” denoting it as a master plant and guide.

Bouso et al. [55] mentioned that psychedelics cause a profound psychological impression, that induces a change in interest and attitude, from less materialistic values to higher open mind and even to mystic-like feelings [55]. Finally, Blainey [18] mentioned that ayahuasca is not a magic panacea if not a master that instructs to the people how to overcome their ego and whatever other sources of suffering in the people [18], whereas Fábregas et al. [56] opined that the ritual of ayahuasca does not appear to be associated with the deleterious psychosocial effects caused by other drugs of abuse [56]. Instead, patients, who suffer drug dependence, can be helped with psychedelic ayahuasca-assisted psychotherapy and other similar medicines [57]. From all the above it is clear that ayahuasca might be the formula for many ailments, as much emotional as physical.

## Social Impact

The social impact of the benefits of ayahuasca worldwide is evident, in any country, rich or poor and inclusive in any social strata. In the past, ayahuasca was used for taking significant decisions of a tribe, for instance for hunting or declaring war, and it was at the center of initiation rites [58]. Nevertheless, the use and interest of ayahuasca have spread beyond of Amazonian tribes. Ayahuasca is being used in countries such as Brazil, Spain, Holland, New Zealand, Australia, United States, and some parts of Asia [3, 59].

In the mid-1990s the use of ayahuasca in occidental cities was popular among people searching for cures and spirituality and therapeutics practices [59]. This phenomenon has been caused by commercial and controversial interesting its use as reflected in different media communications. In the occidental world, ayahuasca has been featured in television programs such as Nip/Tuck and weeds, in which it is a theme of discussion [60]. In Canada, Marl Ellam created a documentary called “Nature of Thing,” which reported on the use of ayahuasca in the community, in psychotherapy, and neurochemistry [61]. Other filmmakers such as Maxi Cohen, an artist from New York City made the film “The Holy Give,” which discussed the roots of the Amazonian people and use of ayahuasca [62].

Despite all this, ayahuasca use have legal limitations. DMT (**1**) is a psychoactive compound in ayahuasca that is under international control since 1971. However, the United Nations Convention on Psychotropic Substances allowed for indigenous peoples to use traditional medicines and sacraments even if those substances are prohibited [3]. Interestingly, in the1980s, some officials began to pay more attention to ayahuasca, mainly in South America [33].

Brazilian Ministry of Health placed ayahuasca on the list of illegal drugs in 1985 [5]. In 2010, the Brazilian government regulated the consumption of ayahuasca by children and pregnant woman due to lack of scientific study about the potentially damaging effects of ayahuasca on the health of children and fetuses, based in the “exercise of parental rights” [63]. Moreover, The International Narcotics Control Board (INCB), in 2010 recommended that the governments be vigilant about the use and abuse of ayahuasca [64].

Nevertheless, Mabit [53] reported about a detoxification center for drug and alcohol addicts [53], which combine old knowledge with contemporary psychotherapeutic practices. This successful method includes the use of plants, psychotherapy and community life and it is recognized by Peruvian authorities [54]. The Takiwasi Center (“the house that heals” in Quechua) [13], is a community in Tarapoto, Peru, that use ayahuasca to treat addiction to cocaine paste [54]. Takiwasi professionals use ayahuasca to help patients overcome their addiction by modifying their “state of consciousness” without damaging themselves [2]. This therapy provided by Takiwasi center has shown excellent results in patients with addictive illness [65].

The spiritual component is essential in these therapies because it offers resilience to someone in treatment which is responding to life stressors; it makes them feel more connected, and to explore their lives can make them more supportable [66]. Ayahuasca has permeated and influence all social and cultural levels. In relation with the illegality in the US of Ayahuasca, Wikelman [41] pointed that it must not be considered as whatever abuse substance, but as a substance that possesses positive cultural influence and beneficial effects in the individuals [41].

## Chemistry

**Fig. 3:**
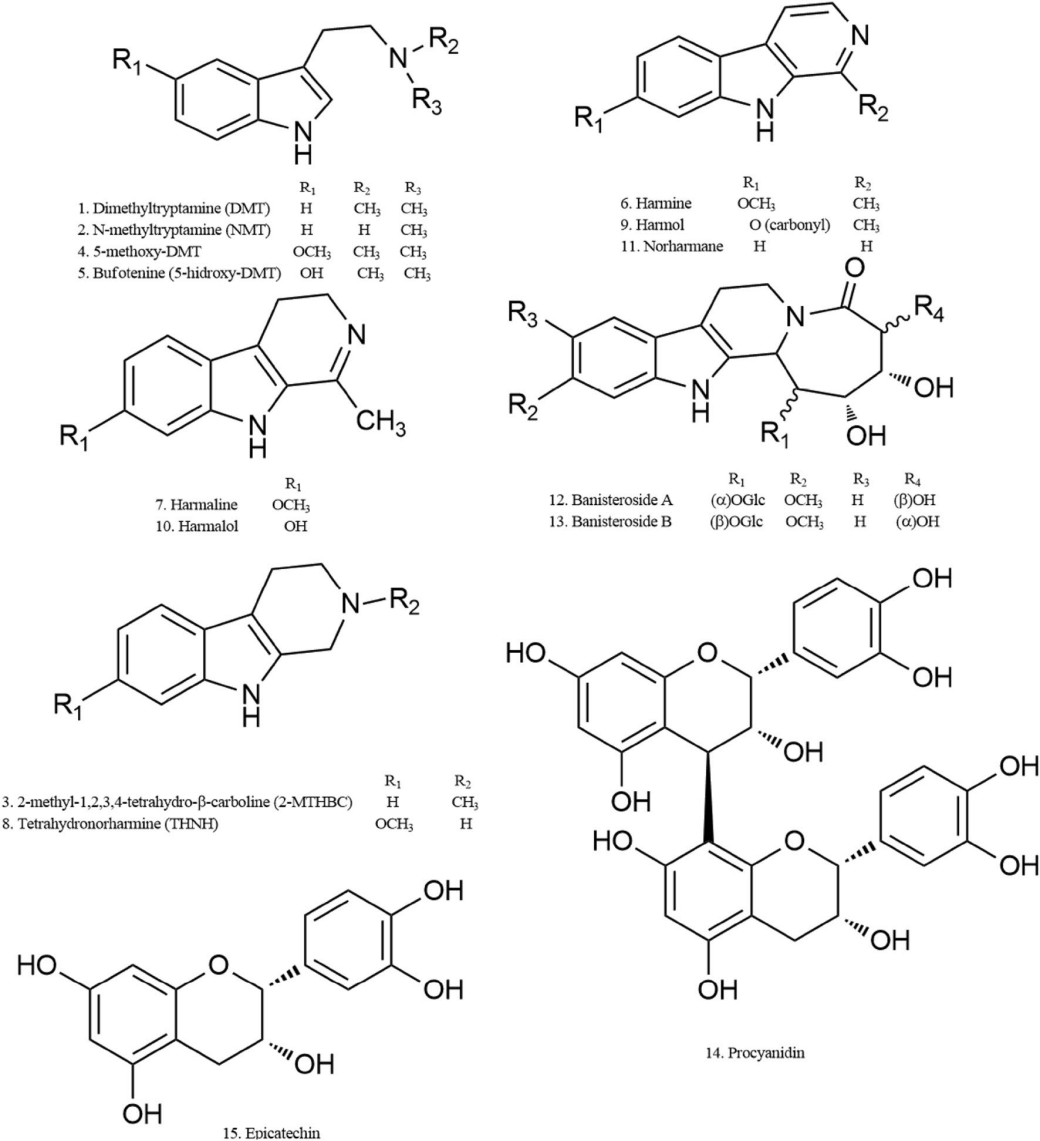
Main compounds in *P. viridis* (**1**-**5**) and *B. caapi* (**6**-**15**)

First isolated alkaloid from *B. caapi* resulted be harmine, reported previously from *Peganum harmala* [67–69]. Other indole alkaloids known as β-carbolines of *P. harmala*, harmaline and tetrahydroharmine, also were identified in *B. caapi* [70–72]. Ayahuasca beverage also contains DMT (**1**) from *P. viridis* leaves, which initially was discovered from *Mimosa tenuiflora*, a compound sub-classified as a simple tryptamine (Fig. 3, **1**). Chemical analysis of ayahuasca beverage using different methods has been driven by a specific scientific interest in the investigation of natural resource, by clinical researches and for the necessity of identifying abuse drugs associated with their components [73]. Techniques used to analyze ayahuasca beverage, including associated metabolomic processes, are enlisted in Table 1.

**Table 1:** Techniques used to analyze ayahuasca beverage

Technique	References
Ambient ionization mass spectrometry (direct analysis in real time-high resolution mass spectrometry, DART-HRMS)	[73]
GC/MS	[74–76]
Solid-phase micro-extraction (SPME)/gas chromatography-ion trap mass spectrometry (GC–IT–MS)	[77]
Liquid chromatography–electrospray ionization-tandem mass spectrometry (LC–ESI–MS/MS)	[78–81, 83, 85]
Gases chromatography with a nitrogen-phosphorous detector	[84]
UPLC chromatography and Q/TOF mass spectrometer (UPLC/ESI-QTOF.MS)	[82, 86]
LC-UV	[87, 89]
Thin layer chromatography	[87]
Capillary electrophoresis (CE) with laser-induced fluorescence (LIF) detection and electrospray ionization-mass spectrometry (ESI–MS)	[88]
NMR^1^H	[90, 93]
NMR^13^C, FTIR and UV/Vis absorption techniques	[91]
Pulse voltammetry with a boron-doped diamond electrode	[92]

*Psychotria viridis* contains DMT (**1**), and lesser N-methyltryptamine (NMT) (**2**) and 2-methyl-1,2,3,4-tetrahydro-beta-carboline (2-MTHBC) (**3**) [79–81]. In combination with other plants, may contain other hallucinogens as 5-methoxy (**4**) and 5-hydroxy-DMT (**5**) (Fig. 1) [84]. *B caapi* contains harmine (**6**), harmaline (**7**) and tetrahydronorharmine (THNH)(**8**) as the major active components and lesser of harmol (**9**), harmalol (**10**), and norharmane (**11)** [79–81, 88]; In addition, structures of *β*-carboline alkaloids: banistenoside A (**12**), banistenoside B (**13**), and procyanidins: procyanidin B2 (**14**) and epicatechin (**15**) from *Banisteriopsis caapi* also have been reported by Wang et al. [87] (Fig. 3). Of these, compounds used as biomarkers are DMT (**1**), harmine (**6**), and harmaline (**7**), which were identified in six typical beverages of ayahuasca, prepared with distinct combinations of *Psychotria viridis*, *Mimosa hostilis*, *Dyplopteris cabrerana*, *Tetrapterys mucronata*, *Banisteriopsis caapi* and *Peganum harmala* among other [72, 75–77, 79, 83, 92–97].

### Biological Effects in Experimental Animals

Research around the world has reported benefits, saying that daily ayahuasca ingestion enhances creative thinking and visual creativity [97]. It has been postulated that ayahuasca may induce the development of self-knowledge, help prevent drug abuse, enable greater self-control and improve social relationships and with the environment [98]. Several biological effects of ayahuasca in experimental animals have been reported.

Ayahuasca daily administered during 30 days at a dose of 120 mg/kg to rats, increased the conditioned fear response and anxiety, which can interfere with the association of emotional events [99]. Lima [100] reported that ayahuasca (2.5 mg/kg) has antidepressant activity, with locomotor activity (44%) and reduced vertical exploration (62.12%) [100]. Respect to the action of ayahuasca in the brain, many studies with similar results have been reported. In Wistar rats, the 5-MeO-DMT was evaluated as antipsychotic drug via functional magnetic resonance imaging (fMRI), showing disrupted cortical activity, altering the discharge rate (86%) in pyramidal neurons, reducing low-frequency cortical oscillations (LFCO); suggesting antipsychotic action [101].

Moreover, the monoamines increased in the amygdala of rats, releasing inhibitory amino acids in the hippocampus, involving changes in monoamines and amino acids concentration [102], like glycine, with possible action on metabolism [26, 27]. In counterpart, ayahuasca exhibited effects on the sexual response, decreasing the number of mounts, intromissions, and ejaculations in Wistar rats [103].

In mice, ayahuasca did not change the neuron bodies number of the analyzed cerebral cortex [104]. Ayahuasca (500 mg/kg) exhibited also therapeutic effects in mice on abuse substance problems, as alcohol [105], probably involving the role modulator of harmine on the dopamine (DA) neurotransmission, evoking DA efflux due to the electrical stimulation of the nucleus accumbens [106]. Harmine was suggested as a molecule that can act against cocaine dependence [107].

In a silico assay, ayahuasca beverage has tested be a novel therapeutic, which can help to understand illness as Alzheimer’s diseases, depression, anxiety, and drug abuse [108]. In this way, ayahuasca influences on neuronal system involve interoception and emotional processing, suggesting that it has antidepressant properties in rats [108], preventing the development of ethanol-induced behavior sensitization without exerting addictive potential, as well as without modifying spontaneous locomotor activity [105].

Also, Wang [88] reported that ayahuasca has epicatechin and procyanidin, which possess antioxidant effect [88]. *B. caapi* was reported with microbicidal activity against *E. coli* (MIC 0.0652 mg/mL; ethanolic extract) [109]. Although several experimental studies have been performed in treating to explain the therapeutic effects of ayahuasca, there are still many questions to answer to understand their effects on human.

In synthesis, the pharmacologists said that the ayahuasca is for the trance or “conscience expansion,” while the tradition said that are “visionary substances” [69]. From this last perspective, an individual may encounter “intelligent spirit beings,” as well as visions and journeys; from biomedicine view, these visions are merely labeled as hallucinations [110]. Thus, the use of ayahuasca is interpreted according to worldview [69]. Ott [111] denominated to the ayahuasca as ‘pharmahuasca’ due to its pharmaceutic relevant properties [111]. Therefore, an approaching that involves both kinds of view should be considered to understand the biological effects of ayahuasca. According to Frecska et al. [112], the therapeutic effects of ayahuasca should be understood from a bio-psycho-socio-spiritual approach [112].

## Biological Effects in Clinical Studies

In human, the effects of ayahuasca have been widely reported, and its use has been associated with multiple pharmacological effects. Thus, Rios and Dobkins [14] investigated the effects of ayahuasca on 20 subjects, who presented illusions, synesthesia, and pseudo-hallucinations [14]. Riba [113] studied in six healthy volunteers the tolerability and psychological effects of ayahuasca, containing 0.5 mg/kg to 1.0 mg/kg of DMT; at least in five subjects induced modified state of awareness with psychedelic effects, modifications in perception and thought processes, qualifying to ayahuasca as an experience pleasant and satisfactory [113]. Also, 28 adolescents that ingested ayahuasca wore healthier and thoughtful than others 28 adolescents not exposed to ayahuasca [114].

Dos Santos in 2011, informed in 11 volunteers, that ayahuasca induces moderate impact on the autonomous nervous system and in the activation of the hypothalamic–pituitary–adrenal axis, increasing prolactin levels, blood pressure, and causing mydriasis [115]. Dos Santos [116] also reported in nine volunteers that ayahuasca could cause vomit, inducing psychotropic effect at a dose of 0.75 mg/kg of DMT, as well as increasing brain electrical activity.

Reports indicate that induced a psychedelic change in the anteroposterior coupling of electrophysiological brain oscillations in humans [117], with the decrease of the frequencies [118]. Ayahuasca in 11 volunteers studied by electroencephalography (EEC) induced effect over the 40 Hz band but did not change the alpha, beta, and theta bands (14–30 Hz) [78, 119]. In contrast, ayahuasca induced in the brain, an acute biphasic effect, changing the alpha and beta band at 2 h after ingested [123]; decelerating the binocular rivalry [121, 122] with hallucinogen effect of gamma oscillations in the visual pathways [123], and modifying the state of consciousness [120, 121]. Riba [123], reported in 18 volunteers that ayahuasca containing 0.85 mg DMT/kg, showed activity in region cortical after 60 min administration, with a decrease in delta and theta power activity, which is the general feature of psychostimulants [123]. Meanwhile, in 18 volunteers have been shown that ayahuasca, decreased the amplitude in P50 (inter-stimulus interval typically of 500 ms, leads to a decrease in the amplitude of the second P50 wave), suggesting a suppressing effect of the drug on normal sensory gating in humans [124].

The effects of chronic ayahuasca ingestion have been also well studied, especially at the central nervous system level. Via functional magnetic resonance imaging (fMRI) in ten volunteers, ayahuasca decreased activity through of the default mode network (DMN), suggesting that ayahuasca decrease the activity of core DMN structures. In 22 members of Santo Daime Church that ingested ayahuasca for around 5.3 years, it was found that structures involved in the DMN and attention/cognitive control include the posterior cingulate cortex (PCC) and precuneus and the medial prefrontal cortex (mPFC) [28, 125]. Besides, ayahuasca in other ten volunteers increased the activation of several occipital, temporal and frontal areas, especially zones closed to the processing of contextual association, memory, intentional prospective imagination and vision [76], with inhibition of the receptor 5TH_2A_, present mainly in the alpha region in the visual network [126].

Osório et al. [127], reported that ayahuasca ingested by six volunteer whit a diagnosis of recurrent depressive disorder and bipolar depression, at a volume 120–200 mL (2.2 mL/kg body weight), during 21 days, exhibits significant acute antidepressant effects, calling to ayahuasca as a fast antidepressant action agent, due to providing a rapid reduction of depressive symptoms [127]. The group of Ribeiro and Dagalarrondo [128] showed the effect of ayahuasca given during 4 weeks (28 volunteers) and 6 months (23 volunteers) to members of Santo Daime and UDV groups [128, 129]. Volunteers showed visual phenomenon, numinosity, peacefulness, reduction of the intensity of psychiatric symptoms, assertively, serenity and vivacity [130].

Ayahuasca in 15 volunteers (1.0 mg/kg, DMT) interacts with neuronal systems, which is central to interoception and emotional processing as well as point to a modulatory role of serotonergic neurotransmission, notably the inhibition of 5TH-_2A_ receptor [31, 131], permitting the restoration serotonin level I in the brain [31]. However, in thirteen healthy subjects of UDV church, ayahuasca decreased the concentration extracellular of 5-HT_2A_ [132] which could explain the results of Santos [133], who reported in nine healthy volunteers from Santo Daime church, that ayahuasca did not decrease the anxiety of the volunteers, who began this study with low levels of anxiety.

Also, ayahuasca mitigated the aggressive personality with extra-sensorial experiences, in a person sentenced to 17 years in prison [131]. It is known, that the activation of the 5-HT_2A_ receptor is associated with aggression in the people [131]. Although the results are promising, the psychological changes should be observed with caution [133], since, in patients with mental disorders as schizophrenia, the DMT has been involved, a molecule present also in ayahuasca drink [134].

On another context, in six volunteers, smoked ayahuasca was more psychoactive that by via oral, this due to that smoked route initially bypasses the liver altogether, and compounds did not suffer an intense metabolism [135]. Also, Callaway [94] in 15 volunteers reported that harmine induces most profound visionary effects than DMT, which presents an accelerated metabolism that involves CYP2D6. Tetrahydroharmine (THH) gave a pharmacokinetic profile independent of harmine [94]. In other 18 volunteers, ayahuasca induced at 1.5 h, perceptual modification and increases in the rating of positive mood and action; the authors suggested that harmine interacts with DMT at gastrointestinal and liver level, which could impact in their metabolism and, in consequence, in their activity [136]. In general, the ayahuasca is promissory to the clinic practical [137], which should represent an incentive to continue with its pharmacological investigation [1].

## Inhibition of the Monoamine-Oxidase (MAO) and Mechanisms Associated with the Biological Actions of Ayahuasca

It is well known that the tryptamines and carbolines compounds are the primary active principles of ayahuasca [54]. The interaction between monoamine oxidase inhibitors (MAOI) and tryptamine hallucinogens is essential, due they are ingested concurrently [138]. In 1967, the Holmsted Lindgren hypothesis of the ayahuasca effect was because to DMT and consequent to MAO inhibition from simultaneous ingestion β-carbolines [113]. Thus, administered orally, DMT is usually destroyed by the liver MAO, but when it is ingested in conjunction with harmaline, harmine, and other β-carbolines, these avoid the effect inhibitory metabolic of the MAO over DMT [69].

In general, β-carbolines work as reversible inhibitors of the A-type isoenzyme of the monoamine oxidase (MAO), with additional selective serotonin reuptake inhibitor (SSRI) effects [112]. Studies indicate that the psychotherapeutic potential of ayahuasca is based mostly on the strong serotonergic effects of tryptamines, reflecting action on serotonin (5-HT) receptors (5-HT_1A_, -_2A_ and -_2C_) as well as the trace amine associated receptors (supposedly TAAR6) [112]. The inhibition of the MAO exerts antidepressant actions due to DMT produces anxiolytic effects through a 5-HT_1A_ receptor agonism [139].

Compounds as 5-methoxy-*N*,*N*-dimethyltryptamine (5-MeO-DMT) are agonist 5-HT_1A_ and 5-HT_2A_ [115]. The 5-MeO-DMT in combination with inhibitors of MAO decrease locomotor activity, inducing delayed of hyperactivity [130–142]. Due to the harmaline inhibits MAO_A_ but not MAO_B_ activity, it has been suggested that ayahuasca beverage can have effect antiparkinsonian as well as other properties to treating other neurodegenerative disorders [143, 144]. Therefore, the MAOI alters the pharmacokinetic and the action of tryptamines, increasing its concentration in the central nervous system and propitiating its interaction with other targets, which could explain the hallucinogen effects of ayahuasca, as well as its adverse effects and toxicity [138].

According to Frecska et al. [112], hallucinations can be interpreted on the bases of receptor–receptor and ligand-receptor interactions, such as “receptor oligomerization,” receptor trafficking,” or biased agonism,” which activate different G proteins resulting in divergent intracellular cascades that mainly involve 5-HT_2a_ receptors and mGluR2 [112]. These investigators reported that the action of DMT and other tryptamines also could be explained through Sigma receptors (Sig-1R), chaperone molecules concentrated in brain, retina, liver, heart, immune system and in many tumor lines, that mediate many signaling pathways, including neuronal differentiation, stress response, oxidative stress, apoptosis, etc. [112]. Therefore, many of the effects observed in ayahuasca can result from its action against oxidative stress and low-grade inflammation via Sig-1Rbe due to the contained of DMT and their tryptamines associated [112]. However, more experimental pieces of evidence are necessaries to explain these associations.

## Toxicity in Experimental Animals

About of the toxicity of ayahuasca, the theme is also controversial. Lima [100] reported in rats that ayahuasca given at 2.5 mg/kg decrease locomotor activity (44%) and vertical exploration (62.12%) [100]; whereas it produces low toxicity, with a LC > 15 mg/kg of DMT, 13.1 mg/kg of harmaline, and 167 mg/kg of harmine) [109]. Ayahuasca, administered at 4 mL/kg in Wistar rats for 14 days, produced flattening and stretching of the vascular smooth muscle cells, changes in the arrangement and distribution of collagen and elastic fibers, an increase of the rate of media thickness to lumen diameter, suggesting arterial wall hypertrophy, a feature of the hypertension [145]. Kawanishi [146] reported that β-carbolines, as harmine and harmaline, induce in mice more tremors/convulsions what others β-carbolines, being β-carbolines most active than tryptamines derivatives [146].

In pregnant rats, the exposure of ayahuasca during gestation produced maternal toxicity, reducing feed intake and maternal weight gain, but only is evident in the necropsy bodies, due to ayahuasca did not provide clinical signs of toxicity [147]. Ayahuasca induced mild anorexia in rats that returned to typical values after 15 days ingested of ayahuasca, suggesting a risk of maternal and developing toxicity [147, 148]. Oliveira et al. [149] also reported that ayahuasca beverage reduces in pregnancy and lactating rats the general anxiety and social motivation of the rat offspring; inclusive, ayahuasca did not induce maternal toxicity at the evaluated conditions as well as did not alter physical and reflexological parameters or body weight, with catalepsy and stereotyped behavior unaltered [149, 150].

Oliveira et al. [148] believe that β-carbolines alkaloids can cross the placental barrier. Therefore, research is necessary around the metabolism of the ayahuasca compounds to determine the adverse effects in the organism and its toxicity in the pregnant woman, a relevant topic due to that in some countries the consumption of ayahuasca by children and pregnant women occur without restrictions.

## Toxicity in Human

In Brazil, the use of ayahuasca by children and pregnant women is legal, due to the Brazilian Civil Code of 2004, that considers that the pregnant women and children that consume ayahuasca fall the domain of the “exercise of parental rights” [64]. Also, at seemly, the effect of ayahuasca in frequent consumers in Peru are longevity, physical vigor, and mental acuity, with ages between 70 and 90 years [10]. Therefore, people that use ayahuasca in syncretic religions for more of 30 years old have not been presented adverse health effect [10], being the ayahuasca, a beverage without toxicity, between the people that regularly consume ayahuasca [93, 94]. Thus, people that drank ayahuasca reported that it did not affect their lives negatively [49]. According to Labate [65], more studies address these issues are necessary.

Bouso [151], reported in 127 ayahuasca users as well as in 115 non-users, the absence of secondary effects as mental performance, life attitudes, personality, and psychopathology, spiritual head and cognition [151]. Barbanoj [152], reported that ayahuasca did not induce any subjectively perceived deterioration on sleep quality after daytime to the consumption of ayahuasca by 22 young, healthy male volunteers [152]. However, ayahuasca harmful effects have been reported, as well as of its components and analogous compounds.

## Cases of Intoxication Associated with the Ingestion of Ayahuasca

Several instances of poisoning for ingesting ayahuasca or their compounds have been reported, some associated with the simultaneous intake with one or various other drugs, as the mushroom *Psilocybe semilanceata*, cannabis, alcohol, and tobacco [153, 154], or overdose, causing a mild disorder (mood disorder episode) to death due to ingestion of dose 10 times higher than the recommended, respectively. In another case, 29 members of the UDV group presented psychotic symptoms associated with ayahuasca ingestion between 1994 and 2007, which was probably due to predisposing psychological features [66].

Another report associated an individual with polar disorders, that presented a manic episode after in a 4-day ingested ayahuasca, without any caution [155]. Also, intoxication by β-carboline alkaloids in a *P. harmala* seeds infusion was reported [156]. Another case happened to a young white male of 25-year-old, who drank ayahuasca before to sleep, but the next morning day was found dead; the medical personnel mentioned that the cause of the dead was hallucinogenic amine intoxication, the case is still unresolved [157]. About of this case, Callaway [158] mentioned that the amount of 5-MeO-DMT presented in the young male as well as the amount that intake a regular indigenous healer was too similar; concluded that probably the young male ingested synthetic material [158]. In the syncretic churches, the amount consumed of ayahuasca ceremony is around 100 mL (82.3 mg/mL), which is ingested orally [159]. Nevertheless, in the samples of ayahuasca seized by the Sao Paulo Police, which is used as recreational drugs, the amount of DMT is higher (820 mg/g), with a good potential for intoxication [160].

## Security in the Use of Ayahuasca

Anderson [160] mentioned that at this moment the practice of ayahuasca is safe, but is necessary the consideration of medical and public safety [11, 160]. Inclusive, Doblin [66] mentioned that substances that as ibogaine and ayahuasca are not addictive, but our culture criminalizes substances that produce non-ordinary states of consciousness [66]. Freeland and Mansbach [67] mentioned the therapeutic effect of ayahuasca in psychopathology, but always exists risks of interactions with psychiatric drugs that could generate potentially dangerous effects [67]. Bouso [55] mentioned that the regular use of psychedelic drugs could potentially lead to changes in brain tissue whereas Wiltshire [153] says that the abuse of natural substances can induce illness or death.

Since information about the safe use of ayahuasca is still scarce and inconclusive, Frecska and Luna [161] suggested that to clarify the adverse effects of hallucinogen; it needs to study as many users as healthy volunteers, those who together could throw significant information about the toxicity and therapeutic effects of ayahuasca [161].

Laqueille and Martins [162] reported that the impulse made by the syncretic churches as well as the poor studies that have not been conclusive for their use in the clinic, inducing that the health authorities banned the use of ayahuasca [162]. Davidson [163] mentioned that scientific community, government, analytical chemist, clinicians as well as drug abusers need to cooperate to research as well as communicate the risk of consuming new psychoactive substances (NPS) [163]. However, as it was suggested by Lanaro [159], it is necessary also to include the cosmovision referred in the rituals of ayahuasca in groups that preserve its traditional use and excluding to the people that consume other drugs, which could confound the security of the ayahuasca use [159].

Of all this, it appears that the use of tryptamine kind compounds with recreational purpose is not safe, because the consumer may miscalculate the dose (overdose) or maybe because of susceptibility phenomena. Being this kind of molecules selling on the internet, which the information about these compounds are exclusively based on commercial purposes [33, 164]. Therefore, the abuse of these substances for recreational and non-religious or pharmacological purposes can trigger tragic events, which should be investigated in depth. Callaway and Grobe [165] mentioned, the need to establish parameters for optimal efficacy and safety to ayahuasca use due to the popularity growth it [165]. Therefore, as was said by Linhart [166], the therapeutic use of ayahuasca can be beneficial, but its use should be cautious [166].

## Conclusions and Perspectives

Traditional ayahuasca beverage has a pharmacological, commercial and spiritual interest, among scientific, believers and government people, around the world. This, thanks to its undeniable current cultural significance, which has been traditionally given in the region of the Amazon until nowadays. Ayahuasca use has spread throughout the world, inclusive in cities with more technology and medical advances. However, its rational therapeutic use still represents a challenge for modern science.

In principle, the recognition of ayahuasca use in rituals should be recognized as an immaterial cultural heritage, such as it was proposed in Brazil and Peru [66]. Next, the principles of “the hoasca project” should be retaken from a multidisciplinary perspective, looking to elucidate the acute and chronic psychological and physiological effects of the ayahuasca, as well as identified its active compounds [10], to finally know its safety profiles and pharmacological interactions with other drugs for a reliable therapeutic use [163], probably through using standardized products. In agreement with Re and Ventura [167], an approach multidisciplinary in the study of the ayahuasca still represents a historic opportunity to create a bridge between scientific and traditional medicine [167]. Whereas from a philosophical point of view, Tupper and Labate [64] predict that the study of ayahuasca and other psychedelic resources can also lead to reflections that relate to the practices and products of science itself [64]. In any case, objectivity and scientific rigor through a bio-psycho-socio-spiritual approach [112] are fundamental.

## References

[1] Trichter S, Klimo J, Krippner S (2009). J. Psychoact. Drugs.

[2] Dobkin MR, Grob CS, Baker JR (2002). J. Psychoact. Drugs.

[3] Tupper KW (2002). Can. J. Edu..

[4] Desmarchelier C, Mongelli E, Coussio J, Giulietti A, Ciccia G (1995). Acta Farm. Bonaer..

[5] MacRae E (1998). Intern. J. Drug Policy.

[6] Prue R (2014). J. Pastoral Care Counsel..

[7] Rivier L, Lindgre JE (1972). Econ. Bot..

[8] Ayala FL, Lewis W (1978). Econ. Bot..

[9] Callaway JC, McKenna DJ, Grob CS, Brito GS, Raymon LP, Poland RE, Andrade EN, Andrade EO, Mash DC (1999). J. Ethnopharmacol..

[10] Loizaga-Velder A, Verres R (2014). J. Psychoact. Drugs.

[11] Langdon EJ, de Rose IS (2012). J. Altern. Emerg. Rel..

[12] McKenna DJ (2004). Pharmacol. Ther..

[13] Anderson BT, Labate BC, Meyer M, Tupper KW, Barbosa PC, Grob CS, Dawson A, McKenna D (2012). Int. J. Drug Policy.

[14] Grob CS, McKenna DJ, Callaway JC, Brito GS, Neves ES, Oberlaender G, Saide OL, Labigalini E, Tacla C, Miranda CT, Strassman RJ, Boone KB (1996). J. Nerv. Mental Dis..

[15] Apud I (2015). Anhropol. Conscious..

[16] Ríos O, Dobkin M (1967). Transcult Psychiatry Res..

[17] Jean EL (2013). Rev. Antropol. Sao Paulo.

[18] Blainey MG (2015). J. Relig. Health.

[19] Shen HH (2015). PNAS.

[20] Campbell CL, Yu R, Li F, Zhou Q, Chen D, Qi C, Yin Y, Sun J (2019). Metab. Syndr. Obes..

[21] Thaiss CA (2018). Science.

[22] Merino H, Arrieta D, Jiménez M, Magos G, Hernández R, Susunaga AC, Hernández E, López NE, Almanza JC, Blancas G, Román R, Alarcon FJ (2014). Nutrients.

[23] Moran S, He X, Chin EL, Tovar AR, Torres N, Slupsky CM, Raybould HE (2017). PLoS ONE.

[24] F. Wang, Dissertation, University of Minnesota, 2015

[25] Mardinoglu A, Shoaie S, Bergentall M, Ghaffari P, Zhang Ch, Larsson E, Bäckhed F (2015). J. Nielsen. Mol. Syst. Biol..

[26] Alarcon FJ, Almanza JC, Blancas G, Angeles S, Garcia R, Roman R, Cruz M (2008). Eur. J. Pharmacol..

[27] Contreras E, Blancas G, Cruz M, Almanza JC, Gomez JH, Ventura JL, Zentella A, Lazzarini R, Roman R, Alarcon FJ (2018). Biomed. Pharmacother..

[28] Doyle R (2012). Anthropol. Conscious.

[29] Espinoza Y (2015). Sex Relat. Ther..

[30] Frecska E (2008). Neuropsychopharmacol. Hung..

[31] Gambelunghe C, Aroni K, Rossi R, Moretti L, Bacci M (2008). Biomed. Chromatogr..

[32] Lowell JT, Adams PC (2016). Soc. Cult. Geogr..

[33] Banard WG (2014). Zygon.

[34] Mercante MS (2013). MANA.

[35] Soler J, Elices M, Franquesa A, Barker S, Friendlander P, Feilding A, Pascual JC, Riba J (2016). Psychopharmacology.

[36] Cakic V, Potkonyak J, Marshall A (2010). Drug Alcohol Depend..

[37] Vélez AC, Pérez AG (2004). Adicciones.

[38] Da Silveira DX, Grob CS, De Rios MD, Lopez E, Alonso LK, Tacla C, Doering-Silveira E (2005). J. Psychoact. Drugs.

[39] Shanon B (2003). J. Mind Behav..

[40] Tatjana JS (2010). Anthropol. Conscious.

[41] Winkelman M (2005). J. Psychoact. Drugs.

[42] Kuypers KP, Riba J, de la Fuente Revenga M, Barker S, Theunissen EL, Ramaekers JG (2016). Psychopharmacol. (Berl).

[43] Cavnar C (2014). J. Psychoact. Drugs.

[44] Wolfson PE (2014). Intern. J. Transpers. Stud..

[45] Harris R (2012). J. Psychoact. Drugs.

[46] Doering-Silveira E, Grob CS, de Rios MD, Lopez E, Alonso LK, Tacla C, Da Silveira DX (2005). J. Psychoact. Drugs.

[47] Doering-Silveira E, Lopez E, Grob CS, de Rios MD, Alonso LK, Tacla C, Shirakawa I, Bertolucci PH, Da Silveira DX (2005). J. Psychoact. Drugs.

[48] Dos Santos RG, Moraes CC, Holanda A (2006). Psicologia: Teoria e Pesquisa Set-Dez.

[49] Kavenská V, Simonová H (2015). J. Psychoact. Drugs.

[50] Kjellgren A, Eriksson A, Norlander T (2016). J. Psychoact. Drugs.

[51] Halpern JH, Sherwood AR, Passie T, Blackwell KC, Ruttenber AJ (2008). Med. Sci. Monit..

[52] Shanon B (2010). Phenom. Cogn. Sci..

[53] Mabit J (2001). Revue Intern. Toxicomanies.

[54] Loizaga-Velder A (2005). J. Psychoact. Drugs.

[55] Bouso JC, Palhano-Fontes F, Rodríguez-Fornells A, Ribeiro S, Sanches R, Crippa JA, Hallak JE, de Araujo DB, Riba J (2015). Eur. Neuropsychopharmacol..

[56] Fábregas JM, González D, Fondevila S, Cutchet M, Fernández X, Barbosa PC, Alcázar-Córcoles MA, Barbanoj MJ, Riba J, Bouso JC (2010). Drug Alcohol Depend..

[57] Sessa B, Johnson MW (2015). Br. J. Psychiatry.

[58] Shanon B (2002). J. Conscious Stud..

[59] Tupper KW (2009). Glob. Netw..

[60] Coutinho T (2013). Rev. Est. Conflito Cont. Soc..

[61] Beyer SV (2012). Stud. Anthropol. Conscious.

[62] Labate BC, Cavnar C (2011). Intern. J. Drug Policy.

[63] Labate BC (2011). J. Psychoact. Drugs.

[64] Tupper KW, Labate BC (2014). Curr. Drug. Abuse Rev..

[65] Labate BC (2010). Anthropol. Conscious.

[66] Doblin R (2013). Curr. Drug Abuse Rev..

[67] Freeland CS, Mansbach RS (1999). Drug Alcohol Depend..

[68] Deshayes P (2002). Psychotropes.

[69] Evans RS (1976). J. Psych. Drugs.

[70] S.R. Evans, A. Hofmann, Plantas de Los Dioses. Origen del uso de los alucinógenos. Ed. Fondo de Cultura Económica. 2ª edn. (VII re-impression 2018), pp 124–136

[71] Tittarelli R, Mannocchi G, Pantano F, Romolo FS (2015). Curr. Neuropharmacol..

[72] Lesiak AD, Musah RA (2016). Forensic Sci. Intern..

[73] Callaway JC, Raymon LP, Hearn WL, McKenna DJ, Grob CS, Brito GS, Mash DC (1996). J. Anal. Toxicol..

[74] Yritia M, Riba J, Ortuño J, Ramirez A, Castillo A, Alfaro Y, de la Torre R, Barbanoj MJ (2002). J. Chromatogr. B Analyt. Technol. Biomed. Life Sci..

[75] de Araujo DB, Ribeiro S, Cecchi GA, Carvalho FM, Sanchez TA, Pinto JP, de Martinis BS, Crippa JA, Hallak JE, Santos AC (2012). Hum. Brain Mapp..

[76] Callaway JC (2005). J. Psychoact. Drugs.

[77] Gaujac A, Dempster N, Navickiene S, Brandt SD, de Andrade JB (2013). Talanta.

[78] Don NS, McDonough BE, Moura G, Warren CA, Kawanishi K, Tomita H, Tachibana Y, Böhlke M, Farnsworth NR (1998). Phytomedicine.

[79] McIlhenny EH, Pipkin KE, Standish LJ, Wechkin HA, Strassman R, Barker SA (2009). J. Chromatogr. A.

[80] McIlhenny EH, Riba J, Barbanoj MJ, Strassman R, Barker SA (2011). Biomed. Chromatogr..

[81] Oliveira CDO, Goncalves GO, da Costa JL, Menck RA, Oliveira DS, Yonanime M (2012). Bioanalysis.

[82] Pichini S, Marchei E, García-Algar O, Gomez A, Di Giovannandrea R, Pacifici R (2014). J. Pharm. Biomed. Anal..

[83] Queiroz MMF, Marti GEF, Queiroz EF, Marcourt L, Castro-Gamboa I, Bolzani VS, Wolfender JL (2015). Phytochem. Anal..

[84] Pires AP, de Oliveira CDR, Moura S, Dorr FA, Silva WAE, Yonamine M (2009). Phytochem. Anal..

[85] Riba J, Mcllhenny EH, Valle M, Bouso JC, Barker SA (2012). Drug Test Anal..

[86] Kowalczuk AP, Lozak A, Bachlinski R, Duszynska A, Sakowska J, Zjawiony JK (2015). Acta Pol. Pharm. Drug Res..

[87] Wang YH, Samoylenko V, Tekwani BL, Khan IA, Miller LS, Chaurasiya ND, Mostafizur MR, Tripathi LM, Khan SI, Joshi VC, Wigge FT, Muhammad I (2010). J. Ethnopharmacol..

[88] Huhn C, Neusüb C, Pelxing M, Pyell U, Mannhardt J, Pütz M (2005). Electrophoresis.

[89] Moura S, Garcia FC, Dizioli CR, Pinto E, Yonamine M (2010). Phytochem. Lett..

[90] Gaujac A, Teixeira SM, Araujo AG, de Andrade SJ, Da Cuhna AP, Mauricio JD, Navickiene S, Bittencourt JA (2013). Microchem. J..

[91] Zhao T, Zheng SS, Zhang BF, Li YY, Blih SWA, Wang CH, Wang ZT (2012). Food Chem..

[92] Svorc L, Cinková K, Samphao A, Stankovic DM, Mehmeti E, Kalcher K (2015). J. Electroanal. Chem..

[93] Callaway JC (2005). J. Psychoact. Drugs.

[94] Callaway JC, Brito GS, Neves ES (2005). J. Psychoact. Drugs.

[95] Stankovic D, Mehmeti E, Svorc L, Kalcher K (2015). Microchem. J..

[96] McIlhenny EH, Riba J, Barbanoj MJ, Strassman R, Barker SA (2012). Biomed. Chromatogr..

[97] Frecska E, Móré CE, Vargha A, Luna LE (2012). J. Psychoact. Drugs.

[98] Lizardo de Assis C, Faria DF, Lins LFT (2014). Psicol. Soc..

[99] Favaro VM, Yonamine M, Soares JC, Oliveira MG (2015). PLoS ONE.

[100] Lima LM, Ferreira SM, Avila AAL, Persazzo FF, Schneedorf JM, Hinsberger A, Carvalho JCT (2007). Phytothérapie.

[101] Riga MS, Soria G, Tudela R, Artigas F, Celada P (2014). Int. J. Neuropsychopharmacol..

[102] de Castro-Neto EF, da Cunha RH, da Silveira DX, Yonamine M, Gouveia TL, Cavalheiro EA, Amado D, Naffah-Mazzacoratti G (2013). World J. Biol. Chem..

[103] Alvarenga TA, Polesel DN, García VA, Matos G, Costa JL, Tufik S, Andersen ML (2014). Behav. Proc..

[104] Santos JC, Almeida VA, Alves DN, Aline ES, da Ré FG, Medeiros GJF, Rossi WCJ, Esteves A (2014). Rev. Neurocien..

[105] Oliveira-Lima AJ, Santos R, Hollais AW, Gerardi-Junior CA, Baldaia MA, Wuo-Silva R, Yokoyama TS, Costa JL, Malpezzi-Marinho EL, Ribeiro Barbosa PC, Berro LF, Frussa-Filho R, Marinho EA (2015). Physiol. Behav..

[106] Brierley DI, Davidson C (2013). J. Psychopharmacol..

[107] O’Connor KA, Roth BL (2005). Life Sci..

[108] Pic-Taylor A, da Motta LG, de Morais JA, Junior WM, Santos AD, Campos LA, Mortari MR, von Zuben MV, Caldas ED (2015). Behav. Proc..

[109] Bussmann RW, Malca-García G, Glenn A, Sharon D, Chait G, Díaz D, Pourmand K, Jonat B, Somogy S, Guardado G, Aguirre C, Chan R, Meyer K, Kuhlman A, Townesmith A, Effio-Carbajal J, Frías-Fernandez F, Benito M (2010). J. Ethnopharmacol..

[110] Gearin AK (2015). Aust. J. Anthropol..

[111] Ott J (1999). J. Psychoact. Drugs.

[112] Frecska E, Bokor P, Winkelman M (2016). Front. Pharmacol..

[113] Riba J, Rodríguez-Fornells A, Urbano G, Morte A, Antonijoan R, Montero M, Callaway JC, Barbanoj MJ (2001). Psychopharmacol. (Berl).

[114] de Rios MD, Grob CS, Lopez E, Silviera DX, Alonso LK, Doering-Silveira E (2005). J. Psychoact. Drugs.

[115] Dos Santos RG, Valle M, Bouso JC, Nomdedéu JF, Rodríguez-Espinosa J, McIlhenny EH, Barker SA, Barbanoj MJ, Riba J (2011). J. Clin. Psychopharmacol..

[116] Dos Santos RG, Grasa E, Valle M, Ballester MR, Bouso JC, Nomdedéu JF, Homs R, Barbanoj MJM, Riba J (2012). Psychopharmacol. (Berl).

[117] Alonso JF, Romero S, Mañanas MA, Riba J (2015). Int. J. Neuropsychopharmacol..

[118] Echenhofer F (2012). Anthropol. Conscious.

[119] Stuckey DE, Lawson R, Luna LE (2005). J. Psychoact. Drugs.

[120] Schenberg EE, Alexandre JF, Filev R, Cravo AM, Sato JR, Muthukumaraswamy SD, Yonamine M, Waguespack M, Lomnicka I, Barker SA, da Silveira DX (2015). PLoS ONE.

[121] Frecska E, White KD, Luna LE (2003). J. Psychoact. Drugs.

[122] Frecska E, White KD, Luna LE (2004). Psychopharmacology.

[123] Riba J, Anderer P, Jané F, Saletu B, Barbanoj MJ (2004). Neuropsychobiology.

[124] Riba J, Rodríguez-Fornells A, Barbanoj MJ (2002). Psychopharmacol. (Berl).

[125] Palhano-Fontes F, Andrade KC, Tofoli LF, Santos AC, Crippa JA, Hallak JE, Ribeiro S, de Araujo DB (2015). PLoS ONE.

[126] Valle M, Maqueda AE, Rabella M, Rodríguez-Pujadas A, Antonijoan RM, Romero S, Alonso JF, Mañanas MA, Barker S, Friedlander P, Feilding A, Riba J (2016). Eur. Neuropsychopharmacol..

[127] Osório FD, Sanches RF, Macedo LR, Santos RG, Maia-de-Oliveira JP, Wichert-Ana L, Araujo DB, Riba J, Crippa JA, Hallak JE (2015). Rev. Bras. Psiquiatr..

[128] Ribeiro PCB, Dalgalarrondo P (2003). J. Bras. Psiquiatr..

[129] Ribeiro PCB, Sales JG, Dalgalarrondo P (2005). J. Psychoact. Drugs.

[130] Ribeiro PCB, Maurício IC, Sales JG, Strassman R, Psychoact J (2009). Drugs.

[131] Riba J, Romero S, Grasa E, Mena E, Carrió I, Barbanoj MJ (2006). Psychopharmacol. (Berl).

[132] Callaway JC, Airaksinen MM, McKenna DJ, Brito GS, Grob CS (1994). Psychopharmacol. (Berl).

[133] Santos RG, Landeira-Fernandez J, Strassman RJ, Motta V, Cruz AM (2007). J. Ethnopharmacol..

[134] Ciprian-Ollivier J, Cetkovich-Bakmas (1997). Schzo. Res..

[135] Riba J, McIlhenny EH, Bouso JC, Barker SA (2014). Drug Test Anal..

[136] Riba J, Valle M, Urbano G, Yritia M, Morte A, Barbanoj MJ (2003). J. Pharmacol. Exp. Ther..

[137] Hrdina V, Merka V, Patocka J (2010). Psychiatrie.

[138] Halberstadt AL (2016). Pharmacol. Biochem. Behav..

[139] Herraiz T, González D, Ancín-Azpilicueta C, Arán VJ, Guillén H (2010). Food Chem. Toxicol..

[140] Halberstadt AL, Geyer MA (2011). Neuropharmacology.

[141] Halberstadt AL, Buell MR, Masten VL, Risbrough VB, Geyer MA (2008). Psychopharmacol. (Berl).

[142] Halberstadt AL, Nichols DE, Geyer MA (2012). Psychopharmacol. (Berl).

[143] Samoylenko V, Rahman MM, Tekwani BL, Tripathi LM, Wang YH, Khan SI, Khan IA, Miller LS, Joshi VC, Muhammad I (2010). J. Ethnopharmacol..

[144] Schwarz MJ, Houghton PJ, Rose S, Jenner P, Lees AD (2003). Pharmacol. Biochem. Behav..

[145] Pitol DL, Siéssere S, Dos Santos RG, Rosa ML, Hallak JE, Scalize PH, Pereira BF, Iyomasa MM, Semprini M, Riba J, Regalo SC (2015). J. Cardiovasc. Pharmacol..

[146] Kawanishi K, Eguchi N, Hayashi T, Hashimoto Y (1994). Pharmacol. Biochem. Behav..

[147] Oliveira CD, Moreira CQ, de Sá LR, Spinosa HS, Yonamine M (2010). Birth Defects Res. B Dev. Reprod. Toxicol..

[148] Oliveira CDO, Queiroz CM, Rose LM, de Souza HS, Yonamine M (2010). Birth Defects Res. B Dev. Reprod. Toxicol..

[149] Oliveira CDO, Queiroz CM, de Souza HS, Yonamine S (2011). Rev. Bras. Farmacogn..

[150] Dos Santos RG (2010). Birth Defects Res. B. Dev. Reprod. Toxicol..

[151] Bouso JC, Gonzáles D, Fondevila S, Cutchet M, Fernándes X, Ribeiro PC, Alcázar-Córcoles MA, Sena WA, Barbanoj MJ, Fábregas JM, Riba J (2012). PLoS ONE.

[152] Barbanoj MJ, Riba J, Clos S, Gimenéz S, Grasa ES (2008). Psychopharmacology.

[153] Wiltshire PE, Hawksworth DL, Edwards KJ (2015). J. Forensic Leg. Med..

[154] Umut G, Küçükparlak I, Özgen G, Türkcan A (2011). J. Psychiatry. Neurol. Sci..

[155] Szmulewicz AG, Valerio MP, Smith JM (2015). Int. J. Bipolar Dis..

[156] Frison G, Favretto D, Zancanaro F, Fazzin G, Davide SF (2008). Forensic Sci. Int..

[157] Sklerov J, Levine B, Moore KA, King T, Fowler D (2005). J. Anal. Toxicol..

[158] Callaway JC, Grob CS, McKenna DJ, Nichols DE, Shulgin A, Tupper KW (2006). J. Anal. Toxicol..

[159] Lanaro R, Calemi DB, Togni LR, Costa JL, Yonamine M, Cazenave SO, Linardi A (2015). J. Psychoact. Drugs.

[160] Anderson BT (2012). Anthropol. Conscious.

[161] Frecska E, Luna LE (2006). Neuropsychopharmacol. Hung..

[162] Laqueille X, Martins S (2008). Ann. Med. Psychol..

[163] Davidson C (2012). Prog. Neuro-Psychopharmacol. Biol. Psychiatry.

[164] De Rios MD, Grob CS (2005). J. Psychoact. Drugs.

[165] Callaway JC, Grob CS (1998). J. Psychoact. Drugs.

[166] Linhart S (2015). Schweiz Z Ganzheitsmed.

[167] Re T, Ventura C (2015). J. Nat. Soc. Phil..

